# Evaluation of the Microstructure and Mechanical Properties of a New Modified Cast and Laser-Melted AA7075 Alloy

**DOI:** 10.3390/ma12203430

**Published:** 2019-10-20

**Authors:** Asmaa M. Khalil, Irina S. Loginova, Andrey V. Pozdniakov, Ahmed O. Mosleh, Alexey N. Solonin

**Affiliations:** 1 Department of Physical Metallurgy of Non-Ferrous Metals, National University of Science and Technology “MISiS”, Leninskiy avenue., 4, Moscow 119049, Russia; loginova@misis.ru (I.S.L.); pozdniakov@misis.ru (A.V.P.);; 2 Department of Mechanical Engineering, Shoubra Faculty of Engineering, Benha University, Shoubra St 108, Cairo 11629, Egypt; 3 Department of Heat Treatment and Physics of Metals, Ural Federal University, Mirastreet, 19, Ekaterinburg 620002, Russia

**Keywords:** Al–Zn–Mg–Cu, transition elements, microstructure, mechanical properties, laser melting, corrosion resistance

## Abstract

The mechanical properties and microstructure of as-cast and homogenized AA7075 were investigated. This alloy was modified by adding transition elements 0.3%Sc + 0.5%Zr, 1%Ti + 0.2%B, and 1%Fe + 1%Ni for use in additive manufacturing applications. After adding Ti + B and Sc + Zr, the structure became uniform and finer with the formation of the Al_3_(Sc, Zr) and TiB_2_ phases. Coarse structures were obtained with the formation of an extremely unfavorable morphology, close to a needle-like structure when Fe + Ni was added. The mechanical properties of the modified alloys were increased compared to those of the standard alloy, where the best ultimate tensile strength (UTS) and yield strength (YS) were obtained in the AA7075-TiB alloy compared to the standard alloy in as-cast and homogenized conditions, and the highest hardness value was provided by Fe + Ni additives. The effect of the laser melting process on the microstructure and mechanical properties was investigated. Single laser melts were performed on these alloys using 330 V and a scanning speed of 8 mm/s. During the laser melting, the liquation of the alloying elements occurred due to non-equilibrium solidification. A change in the microstructures was observed within the melt zone and heat-affected zone (HAZ). The hardness of the laser-melted zone (LMZ) after adding the modification elements was increased in comparison with that of the standard alloy. Corrosion testing was performed using a solution of 100 mL distilled water, 3.1 g NaCl, and 1 mL HCl over 5, 10, and 30 min and 1 and 2 h. The corrosion resistance of the alloy modified with FeNi was low because of the non-uniform elemental distribution along the LMZ, but in the case of modification with ScZr and TiB, the corrosion resistance was better compared to that of the standard alloy.

## 1. Introduction

Ultra-high-strength 7xxx alloys are generally utilized in transportation, aerospace applications, corrosion resistance applications, and when good welding properties are required [1,2,3,4]. The disadvantages of these alloys are due to them containing several alloying elements; the received solid structure shows differences in chemical composition from the center of the dendrite to the edges [5]. Alloying elements and contaminations form coarse eutectic structures and arrange in the dendrite and inter-dendritic boundaries during the solidification process. These inconveniences lead to bad castability and high crack sensitivity during hot rolling. The addition of minor elements enhances the metallurgical state of the 7xxx series [6]. Because of casting problems like hot cracking sensitivity, its applications have been limited to wrought products [7]. If the castability is improved, the application fields could be expanded greatly. The properties of these alloys are determined by the precipitation of alloying elements that dissolve in the homogenization process. These precipitates’ phases are accountable for the uniquely high strength of the alloys, which are formed during a variety of heat treatments. The formation of the MgZn_2_ phase in the AA7075 aluminum alloy is severely affected by the Zn–Mg ratio. By increasing the Zn–Mg ratio, the formation of the MgZn_2_ compounds is increased, and the Copper concentration also affects the formation of the new phases of Al_2_CuMgZn [8]. For cast AA7075 to be used in high-temperature applications, it must have a eutectic structure with thermal stability at temperatures of ≥600 °C and precipitate particles of the second phase, which have thermodynamic stability at the service temperature. AA7075 is known as a non-weldable alloy due to its poor solidification microstructure and pore formation in the fusion zone (FZ). After the discovery of the Friction Stir Welding (FSW) process, aluminum alloys that were considered un-weldable were observed to be weldable by FSW; moreover, the resulting welded joints have very good mechanical properties [9]. It has been noticed that the total amounts of Zinc, Magnesium, and Copper in this alloy control the alloy’s characteristics and weldability. Al–Zn alloys with an amount of ≥9 wt % have high strength but with bad weldability [10,11]. Pore formation is a significant problem in laser beam welding (LBW) of the 7xxx series. Gas porosity forms due to a small amount of gas entering the laser-melted zone (LMZ). Due to the high solubility of H_2_ in liquid Al, H_2_ is the main source of pore formation in the alloy. The formation of H_2_ starts from the filler and base materials as an oxide or impurities, then is dissolved in the bulk material [12]. On the other hand, the presence of keyhole porosity occurring due to the keyhole instability during LBW appears in different forms: Necking, swelling, and collapsing. The main reason for the keyhole porosities is the non-uniform vaporization of the fickle alloying elements such as Zn and Mg with different vapor pressure [12]. Hot cracking is the second problem during LBW of these alloys. It can be observed in two different forms—solidification cracks in the LMZ or liquidation cracks in the heat, affected zone (HAZ)—but usually forms in the LMZ. Hot cracking forms due to the low melting grain boundary eutectics with the presence of critical stresses [13]. The chemical composition of the alloy has a big influence on the solidification range. By using different welding parameters, the resulting stresses and the heat input can be affected. To improve the weldability of this alloy, we should reduce the crystallization cracks and pore formation by adding the alloying elements as a modification and inducing grain refinement.

Grain refinement can be achieved in three ways: Chemical, mechanical, and thermal. The most usable way is chemical, which occurs by adding master alloys, preventing grain growth, and enhancing nucleation [14,15,16,17,18]. The most popular grain refiners used in Al and its alloys are Al–Ti–C, Al–Ti, Al–Zr, Al–Sc, Al–Ti–B, Al–Sr, and Al–B [19,20,21,22,23,24]. The best mechanical properties are obtained by a fine equiaxed grain structure. The other advantages of grain refinement are hot tearing resistance, good surface finish, high toughness, good machinability, and high yield strength [25,26]. The main reason for using grain refinement elements in Al alloys is to obtain a fine equiaxed grain structure, which enhances the mechanical properties, reduces liquation and hot crack sensitivity, and improves fluidity. The most popular grain-refining compound used in Al alloys is Al–Ti–B, which consists of soluble Al_3_Ti and insoluble TiB_2_ particles, which are often credited with the grain refining effect. Fe and Ni are usually added into the aluminum alloys in small concentrations to obtain refractory aluminides containing Fe and Ni, which increase the heat resistance of the alloys. Higher concentrations of Fe and Ni in aluminum alloys lead to the formation of excess phases of crystallization origin of unfavorable morphology, which greatly reduces the ductility. However, if we consider these alloys as promising materials for additive technologies, the concentration of alloying elements can be increased due to the higher cooling rate [27,28,29]. Ni has a good effect on the mechanical properties and microstructure of Al alloys, whereby adding Ni to pure Al forms Al_3_Ni through eutectic reaction and increases the hardness of the alloy [30]. Additions of Zr and Sc help to develop new materials for lightweight structures with good welding performance, excellent mechanical properties, creep, and desirable corrosion resistance. The increase in strength by additions of Zr and Sc is mostly connected with the primary Al_3_(Sc, Zr) particles formed during solidification [31,32]. Homogenization annealing is carried out after alloy casting and plays an important role in preventing micro-segregation and dissolving nonequilibrium eutectic particles formed during the casting process [33,34].

Therefore, in this work we aimed to investigate the influence of different alloying elements on the microstructural behavior and mechanical properties in the as-cast and homogenized annealing states of Al–Zn–Mg–Cu alloys and their effect on the laser melting and corrosion behavior of these alloys to improve the laser weldability of these alloys for use in additive manufacturing.

## 2. Materials and Methods

The selected alloy for this work, Al–Zn–Mg–Cu (AA7075), alloyed with 4 different alloying elements (transition elements), was obtained by the standard method of casting into a copper water-cooled mold; the chemical compositions are listed in Table 1. The term AA7075-standard refers to the standard as-cast Al–Zn–Mg–Cu, and AA7075-ScZr, AA7075-TiB, and AA7075-FeNi referred to the as-cast alloy with different alloying elements added, such as Ti, B, Fe, Ni, Sc, and Zr, using master alloys Al–5%Ti–1%B, Al–10%Fe, Al–3.5%Zr, Al–20%Ni, and Al–2%Sc. Homogenization annealing at 460 °C for 3 h was carried out in a Nabertherm electric furnace (Nabertherm, Lilienthal, Germany) with a temperature maintenance accuracy of ± 5 °C. The investigated samples were polished with special equipment, a Struers “Labopol-5” (Struers APS, Ballerup, Denmark) automatic polishing machine, using silicon carbide (SiC) paper with numbers P320, P600, P800, P1200, P2400, and P4000, applying water as a lubricant to the abrasive surface. Then, oxidation of the polished surface was done with a 10% electrolyte (saturated solution of H_3_BO_3_ in HF) in distilled water at 18 V. The microstructural features were observed under polarized light using an Axiovert 200 MMAT (Carl Zeiss, Oberkochen, Germany) optical light microscope (LM) The microstructure and phase composition of the investigated alloys were examined using a Tescan-VEGA3 (Tescan Brno s.r.o., Kohoutovice, Czech Republic) scanning electron microscope (SEM). The calculation of the average grain size and its analysis were carried out using the linear intercept method.

Hardness results were obtained using a HVD-1000AP micro Vickers (Germany Wolpert Wilson Instruments, Aachen, Germany,) tester machine with a load of 49 N and HV5 for 15 s. Microhardness values of the laser-melted area were obtained by a Vickers hardness testing with a load of 500 g and a shutter speed of 10 s. Tensile results were obtained using a Zwick/Roell Z250 (Zwick/Roell, Kennesaw, GA, USA) all-round series-testing machine; the strain rate was 4 mm/min, and the standard deviation from the mean value was within 2 to 4 MPa of the measured values. Differential scanning calorimetry (DSC) analysis was performed on a Setaram Labsys (SETARAM Instrumentation, Caluire, France) calorimeter in an Argon atmosphere, with a heating rate of 5 °C/min. Studies were conducted in the temperature range of 20 to 1000 °C

Laser melting was performed using a pulse-periodic laser welding machine MUL-1-M-200 (OOO Latikom, Moscow, Russia) equipped with an Nd:YAG laser which operates at a wavelength of 1064 nm under protective Argon gas with a voltage of 330 V, pulse duration of 14 ms, an overlap of 0.8 mm, and a laser scanning speed of 8 mm/s. Corrosion testing was performed using 100 mL distilled water + 3.1 g NaCl + 1 mL HCl solution over different times of 5, 10, and 30 min and 1 and 2 h accumulating.

## 3. Results and Discussion

### 3.1. As-Cast Microstructure

**Figure 1:**
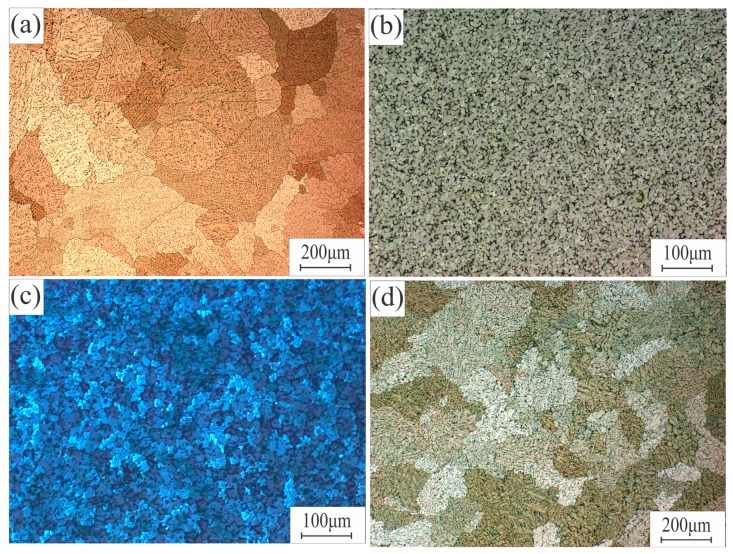
Typical microstructure of the Al–Zn–Mg–Cu alloy: (**a**) AA7075 standard, (**b**) AA7075-ScZr, (**c**) AA7075-TiB, (**d**) AA7075-FeNi.

**Figure 2:**
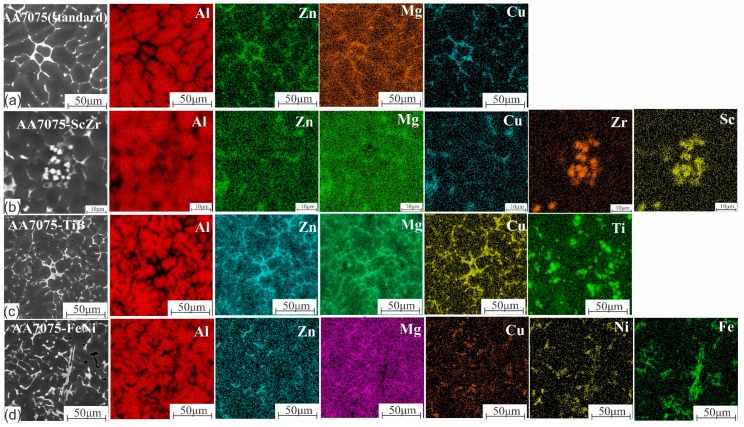
Maps of the elemental distributions of the investigated alloys in the as-cast state: (**a**) AA7075 standard, (**b**) AA7075-ScZr, (**c**) AA7075-TiB, (**d**)AA7075-FeNi.

Figure 1a presents the microstructure of the as-cast standard alloy, which contains primary dendrites of the Al-rich solid solution with a uniform structure with an average grain size of 272 ± 20. Maps of the element distributions of the investigated alloys are shown in Figure 2a–d. For AA7075-standard in Figure 2a, Al dendritic cells were Al-rich with small amounts of elements Zn, Mg, and Cu. However, in the inter-dendritic regions, it can be observed that the eutectic phases were formed from Zn, Mg, and Cu. Adding 0.5% Zr and 0.3% Sc changed the coarse-grained dendritic structure to a limitingly fine nondendritic structure, shown in Figure 1b, with a grain size of 11 ± 2 µm. The presence of Zr and Sc can be observed in Figure 2b as a result of the formation of the primary aluminide Al_3_(Sc, Zr) particles and the eutectic phase of Zn, Mg, and Cu in the inter-dendritic areas.

Adding the master alloy of Al–5Ti–1B resulted in very fine and uniform grains (shown in Figure 1c) due to the effect of the soluble Al_3_Ti as a grain refining agent, which led to a uniform distribution of the liquid phase in the effective crystallization interval with the presence of the insoluble TiB_2_ particles (Figure 2c). The presence of these phases changed the character of the crystallization of the alloy, and the average grain size was 23 ± 2 µm.

In 7075-FeNi, coarse structures were obtained with average grain size 316 ± 15 µm; the formation of the eutectic phases of FeNi, Al_3_(Ni, Fe), and Al_3_Ni crystals, which have an extremely unfavorable morphology (close to a needle-like structure), and other elements were observed in the inter-dendritic areas as a eutectic phase, as shown in Figure 2d.

### 3.2. Microstructures after Homogenization Annealing

**Figure 3:**
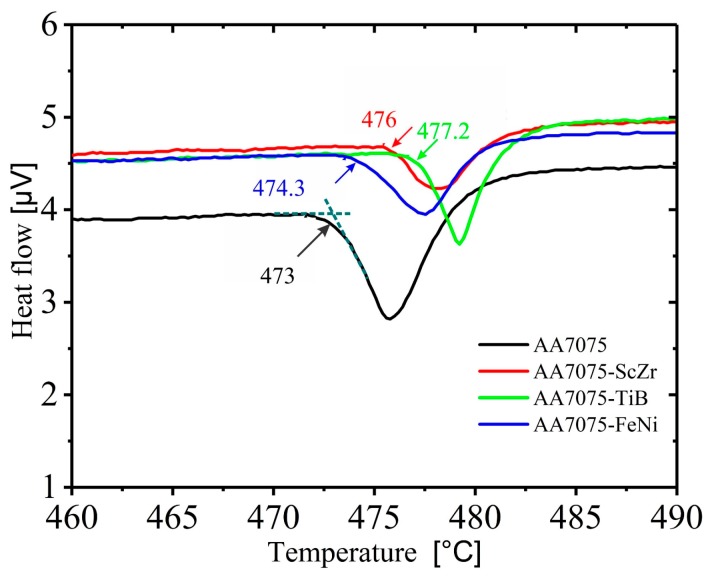
DSC analysis for the investigated alloys.

The observed changes in the structure and shape are due to the movement of the molten metal and different temperature distributions in the liquid pool during casting along the ingot, thus homogenization annealing for ingots should homogenize the grain structure and eliminate the formation of the non-equilibrium eutectic phases. Figure 3 shows the heating DSC curves of the investigated alloy samples cut from as-cast ingots; the heating rate of the samples was 5 °C /min. A suitable homogenization annealing temperature of 460 °C was selected from the DSC analysis. The temperature of the non-equilibrium solidus for the AA7075 standard alloy was 473 °C; when the elements Sc + Zr, Ti + B, and Fe + Ni were added, the temperatures of the non-equilibrium solidus for the three resulting alloys were increased by 1–4 °C compared to that of the standard alloy.

**Figure 4:**
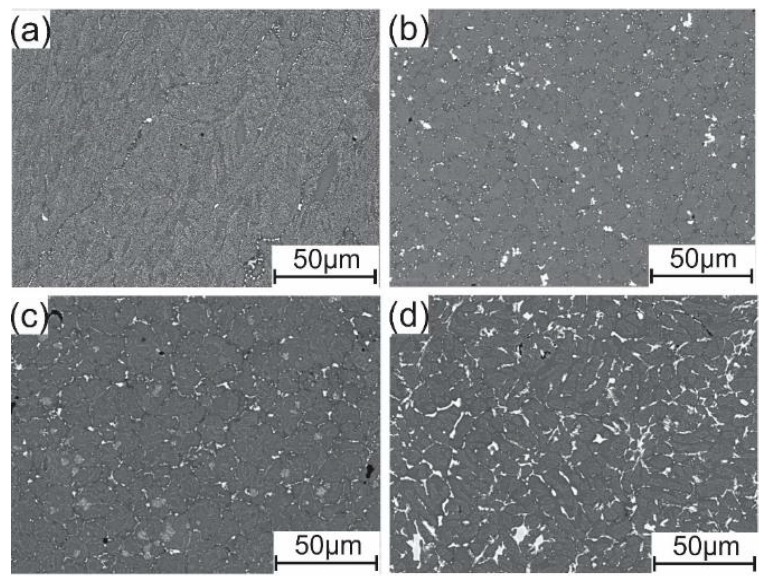
SEM microstructure of Al–Zn–Mg–Cu alloy after homogenization annealing at 460 °C for 3 h: (**a**) Standard alloy, (**b**) with the addition of Sc and Zr, (**c**) with the addition of Ti and B, and (**d**) with the addition of Fe and Ni.

During the homogenization annealing treatment at 460 °C for 3 h, in AA7075-standard, the non-equilibrium eutectic phases formed during the casting were dissolved into the Al matrix and formed a saturated solid solution, and the structure became finer and more homogenous, as shown in Figure 4a. In the cases of the alloys with other elements Sc, Zr, Ti, and B, the formed phases Al_3_(Sc, Zr) and TiB_2_ were uniformly distributed (Figure 4b,c). In the case of adding Fe and Ni, the eutectic phase dissolved in Al solution, Fe and Ni were less soluble in Al, eutectic phases were present in the intermetallic phases, and the Al_3_Ni phase became smaller, as displayed in Figure 4d.

### 3.3. Mechanical Properties in As-Cast and Homogenized Annealing Conditions

The ultimate tensile strength (UTS) and elongation (δ) results of the as-cast and homogenized annealed alloys are summarized in Table 2. The UTS values of the as-cast alloys after adding the alloying elements were increased compared to that of the standard alloy. Adding 0.3% Sc + 0.5% Zr increased the UTS 1.8 times, and elongation was increased by 10.4% because of the dissolving of the non-equilibrium phases into the Al matrix and the formation of the saturated solid solution. When adding 1% Ti + 0.2% B, the UTS was increased 1.8 times, and elongation was increased by 5.3% because of the formation of Al_3_Ti, causing grain refinement. The relation between adding the grain refining elements and high yield strength (YS) can be summarized as small grains obstructing the dislocations and the YS thus becoming higher. After adding 1% Fe + 1% Ni, the UTS and elongation were increased 1.4 times and 1.3%, respectively, due to the effect of the formation of Al_3_Ni through the eutectic reaction; the small effect on elongation was because of the unfavorable morphology, close to a needle-like structure. These results showed that the addition of these elements was useful in increasing and enhancing the mechanical properties of the standard alloy.

After homogenization annealing treatment at 460 °C for 3 h in the AA7075-standard, the dissolving of the non-equilibrium eutectic forming elements Zn, Mg, Cr, Mn, and Cu into the Al matrix and formation of a saturated solid solution led to decreased UTS and YS and increased ductility. In the case of AA7075-ScZr, there was a decrease in the UTS and YS because the Al_3_(Sc, Zr) particles became larger, and these particles stopped the movement of the dislocations, also leading to decreased ductility. In AA7075-TiB, the decrease in the UTS and YS, which was observed after homogenization and the increase in elongation, were because of the same mechanism which occurred in the standard alloy. Finally, in AA7075-FeNi, the UTS and YS were decreased due to dissolving of the eutectic phase in the Al solution, the lower solubility of Fe and Ni in Al, and the presence of eutectic phases in intermetallic phases, but the increase in elongation was because of the spherical shape of Al_3_Ni after the effect of homogenization annealing.

**Figure 5:**
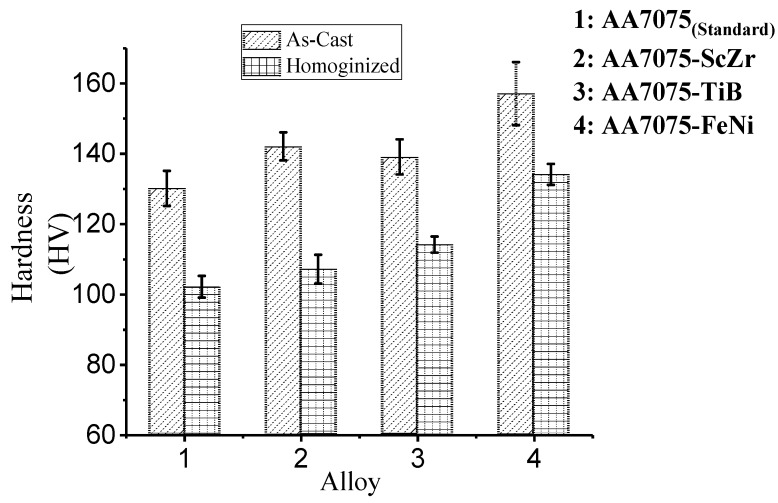
The Vickers hardness results of investigated alloy in as-cast and homogenized conditions.

The Vickers hardness results of the standard and modified alloys in as-cast and homogenized states are plotted in Figure 5. For the standard alloy, the result was 130 ± 5 HV; adding (Sc + Zr) and (Ti + B) increased the hardness values to 142 ± 4 HV and 139 ± 5 HV, respectively, due to the grain refinement, the decreasing of dendrite, and dispersed precipitates of Al_3_(Zr, Sc), and the insoluble TiB_2_ particles, which inhibited the movement of the dislocations. Finally, adding a combination of Fe and Ni led to increased hardness up to 157 ± 9 HV because of the Al_3_Ni phase.

The hardness values after the homogenization annealing were decreased by 0.78, 0.75, 0.82, and 0.87 for the AA7075-standard, AA7075-ScZr, AA7075-TiB, and AA7075-FeNi, respectively, compared to the hardness values in the as-cast condition. This decrease was connected with the dissolving of the non-equilibrium eutectic forming elements Zn, Mg, Cr, Mn, and Cu into the Al matrix and the formation of a saturated solid solution. In the case of AA7075-ScZr, the decrease was because, during annealing at 460 °C, the Al_3_(Sc, Zr) particles became larger, and these particles stop the movement of dislocations.

### 3.4. Laser Melt Zone Microstructure

**Figure 6:**
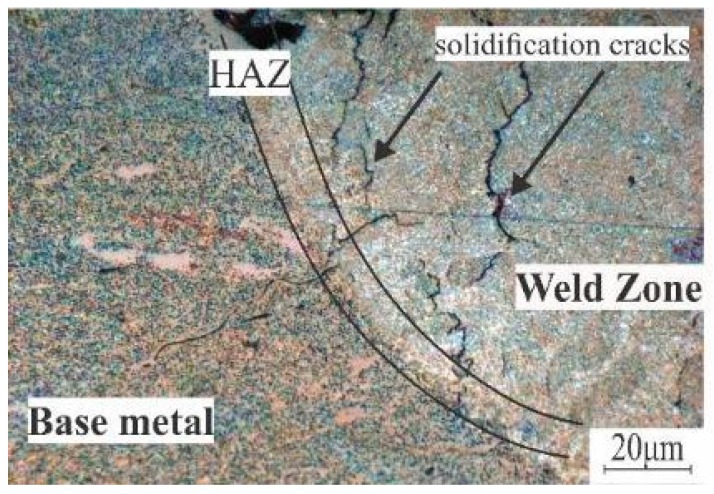
Laser-melted zone (LMZ) of the as-cast AA7075-standard.

Figure 6 shows the effect of laser melting on the as-cast AA7075 standard. Three zones can be observed: Base metal (BM), the heat-affected zone (HAZ), and the laser-melted zone (LMZ). The formation of crystallization (solidification) cracks was found. Cracks were located along the boundaries of equiaxed grains, which started from the base metal. One of the main reasons for crack formation was the melting of the non-equilibrium eutectic phases in the inter-dendritic spaces during the laser melting. The other reason was the high tendency alloy to form crystallization cracks due to the wide effective crystallization range.

**Figure 7:**
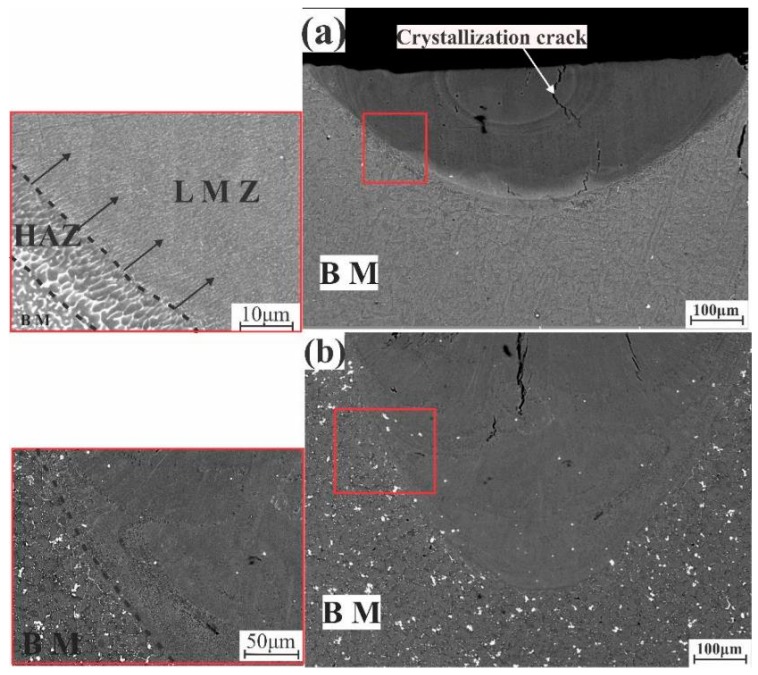
SEM images of the laser-melted zone (LMZ) after homogenization annealing at 460 °C for 3 h: (**a**) AA7075-standard, (**b**) AA7075-ScZr.

**Figure 8:**
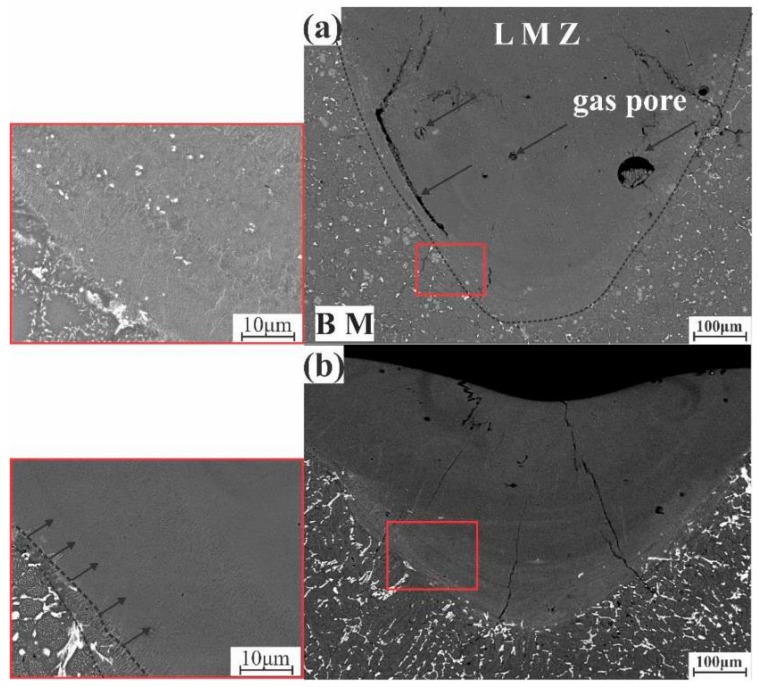
SEM images of the LMZ after homogenization annealing at 460 °C for 3 h: (**a**) AA7075-TiB, (**b**) AA7075-FeNi.

The microstructures after laser melting of the modified alloys and homogenized annealing can be observed in Figure 7 and Figure 8. In AA7075-standard, as shown in Figure 7a, the structure was divided into three zones: The BM, HAZ, and LMZ. The HAZ had a eutectic structure with a columnar shape, which was almost the same as the BM but with a slightly more elongated shape, and these grains had a random crystal orientation. During solidification, the grains in the LMZ started to grow from these randomly oriented grains. Non-dendritic equiaxed grains appeared along the fusion line in the LMZ with a direction parallel to the opposite direction of the laser heat flow (plotted arrows). At the center of the LMZ, there was the appearance of a fine zone in which the structure changed from elongated to fine equiaxed grains. These fine structures were formed due to the high cooling rate during solidification.

In AA7075-ScZr, the boundary between the BM and LMZ was clear, as shown in Figure 7b. The uniform and fine structure can be observed. This grain refinement resulted from the presence of primary Al_3_(Zr, Sc) particles, which formed during the alloy solidification and became the nuclei for the crystallization of the Al solid solution.

In the case of AA7075-TiB, clarified in Figure 8a, it has a very fine and uniform structure in the LMZ. TiB2 particles with little gas pores were formed. During solidification, gas bubbles could not go out from the LMZ forming gas pores.

In AA7075-FeNi, during laser processing, the coarse structure and eutectic crystal were remelted, causing the formation of eutectic intermetallic phases around the dendrites. Structure formation started from the boundary of the BM to the surface in the opposite direction to the heat flow (plotted arrows) shown in Figure 8b. Crystallization cracks were observed in this alloy more than in the other alloys. Fe and Ni did not act as a modifying element during solidification.

### 3.5. Elemental Distribution in the LMZ

**Figure 9:**
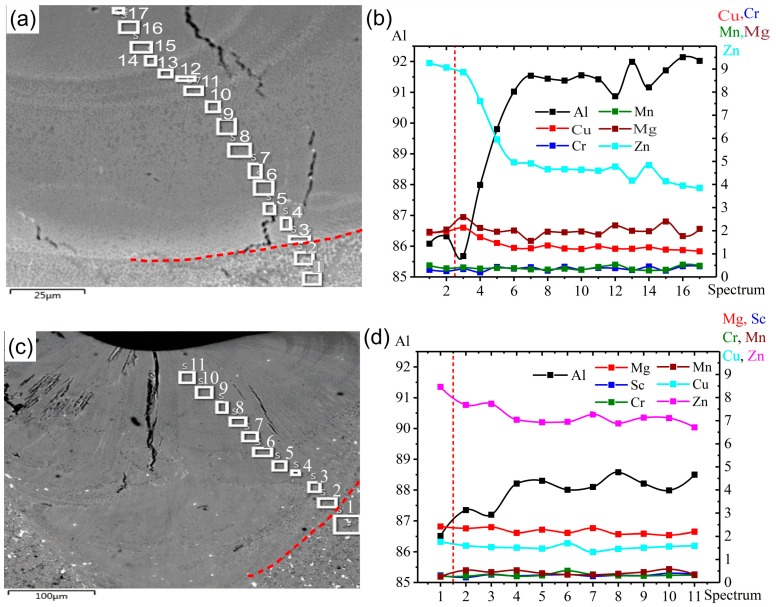
The concentration of the elements at different points along the LMZ after homogenization annealing at 460 °C for 3 h: (**a**,**b**) AA7075-standard, (**c**,**d**) AA7075-ScZr.

**Figure 10:**
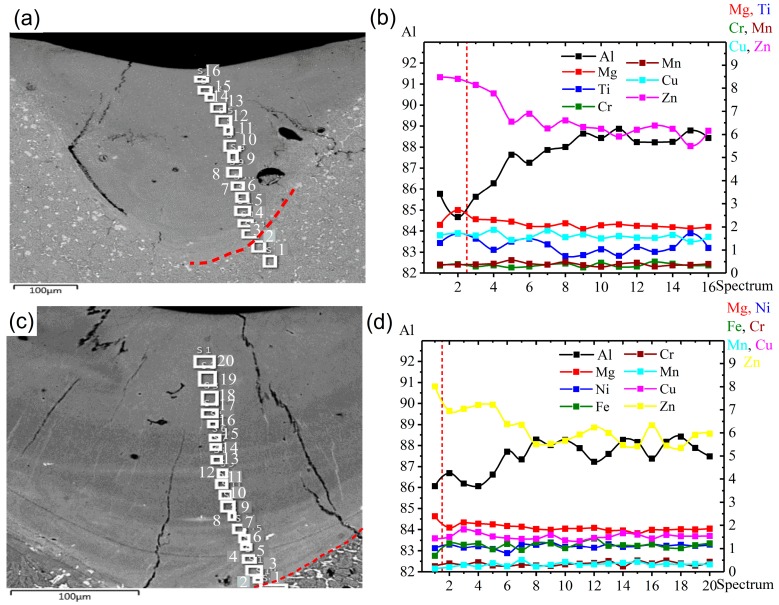
Concentration of elements at different points along the LMZ after homogenization annealing at 460 °C for 3 h: (**a**,**b**) AA7075-TiB, (**c**,**d**) AA7075-FeNi.

The microstructural analysis via SEM showed that at the center and boundary of the welded area, some lines were formed with a bright color, and the elemental distribution analysis of this area showed that these formed lines contained a higher amount of Zn. The formation of these lines related to the liquation effect, as shown in Figure 9 and Figure 10. During laser melting, the liquation of the alloying elements occurred due to non-equilibrium solidification, which led to different concentrations of elements in the LMZ in the AA7075-standard, as shown in Figure 9a,b. Zn and Mg were recorded at higher concentrations near the boundary of the LMZ (at Point 3) due to the precipitation of MgZn_2_ and the difference in temperature between the LMZ and BM during the solidification. Then the concentrations of Zn and Mg increased at Points 12 and 14 again due to the difference in temperature between the liquid and the previous layer and the formation of MgZn_2_. The decrease in Zn and Mg, which occurred in the LMZ, was due to the evaporation effect from high temperatures during laser melting, and other elements were uniformly distributed throughout the LMZ. In AA7075-ScZr, it was noticed that there was a decrease in Zn and Mg in the LMZ compared with the BM because of the evaporation effect at high temperatures. Along the LMZ, all other elements were uniformly distributed, as shown in Figure 9b,c.

In AA7075-TiB, as shown in Figure 10a,b, there was no large difference in the distribution of elements along the LMZ, and they were uniformly distributed due to the effect of Ti and B as refining elements. Finally, in AA7075-FeNi, severe liquation of the elements was observed, and the concentration of Zn and Mg was not uniform along the LMZ with peaks at Points 5, 7, 12, and 16, shown in Figure 10c,d. Thus, the main reason for grain refinement with the addition of Zr + Sc and Ti + B elements was a decrease in the liquation effect due to the heterogeneous nucleation and growth of the crystallizing phases in the presence of many crystallization centers. With the addition of Fe + Ni, the same results with the standard alloy were observed. The distribution of Zn and Mg was unevenly in LMZ due to the evaporation effect during laser process. Fe and Ni did not modify the grain structure, as was shown in Figure 1d, and there was epitaxial-type crystal growth.

### 3.6. Microhardness Results

**Figure 11:**
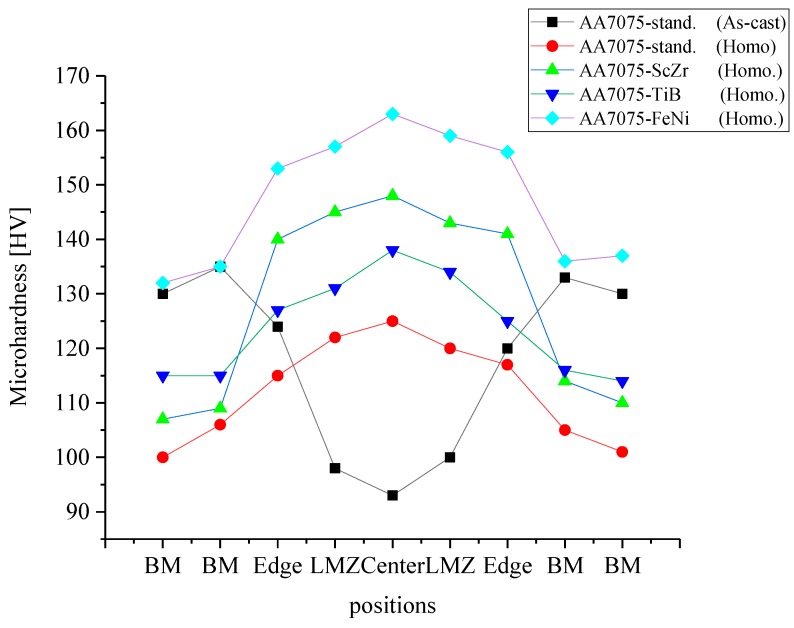
Microhardness results of alloys after laser processing along the base metal (BM) and LMZ.

From the Vickers microhardness values for AA7075-standard in the as-cast state after laser melting, the hardness decreased gradually from a maximum value in the zone of the base metal to a minimum value in the weld zone. The lower hardness in the WZ was because of the evaporation of the strengthening elements, which prevented the formation of the strengthening precipitates and the presence of non-equilibrium eutectic phases. However, after homogenization annealing, the microhardness in the center of LMZ was increased to 125 HV from 100 HV in the BM. With other alloys, the microhardness was increased in the center of the LMZ compared to the BM, as shown in Figure 11, due to the modification elements, the dissolving of the non-equilibrium eutectic phases, and the formation of new phases, which enhanced the mechanical properties of the melting pool.

### 3.7. Corrosion Behavior

**Figure 12:**
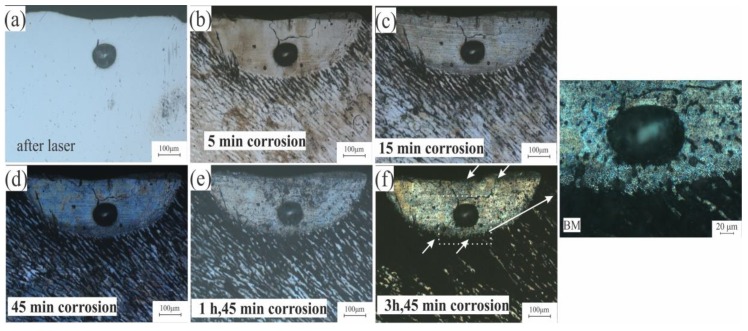
Intergranular corrosion of AA7075-Standard after (**a**) laser, (**b**) 5 min, (**c**) 15 min, (**d**) 45 min, (**e**) 1 h and 45 min, and (**f**) 3 h and 45 min.

**Figure 13:**
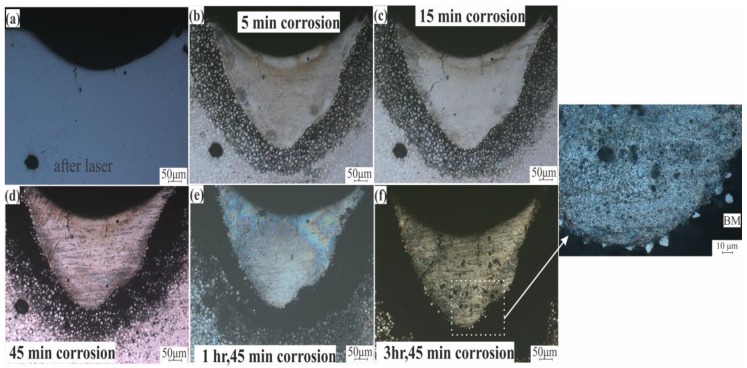
Intergranular corrosion of AA7075-ScZr after (**a**) laser, (**b**) 5 min, (**c**) 15 min, (**d**) 45 min, (**e**) 1 h and 45 min, and (**f**) 3 h and 45 min.

**Figure 14:**
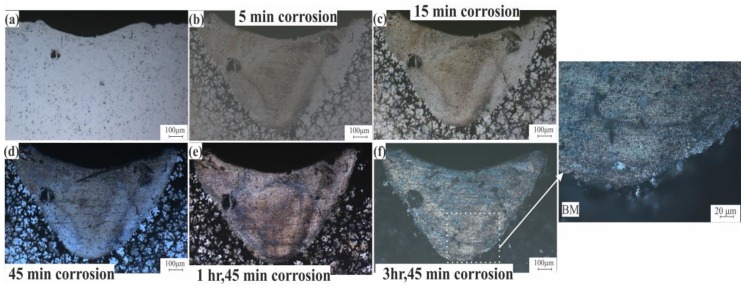
Intergranular corrosion of AA7075-TiB (**a**) laser, (**b**) 5 min, (**c**) 15 min, (**d**) 45 min, (**e**) 1 h and 45 min, and (**f**) 3 h and 45 min.

**Figure 15:**
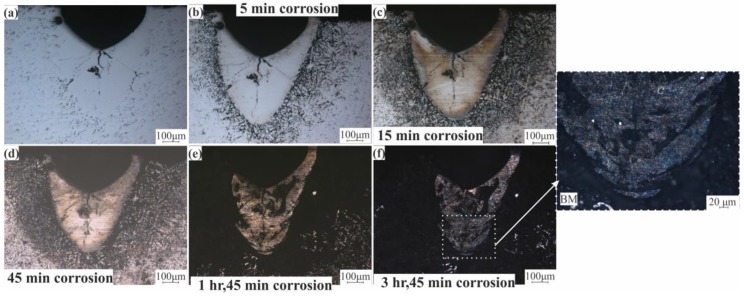
Intergranular corrosion of AA7075-FeNi after (**a**) laser, (**b**) 5 min, (**c**) 15 min, (**d**) 45 min, (**e**) 1 h and 45 min, and (**f**) 3 h and 45 min.

Intergranular corrosion testing for standard and modified alloys was done to check the effect of the liquation phenomena on the corrosion behavior on these alloys. Figure 12a shows the LMZ of the AA7075-standard after laser processing. After 5 to 45 min of testing, there was no difference in the LMZ. The effect occurred only on the BM because of the higher concentration of Zn in this area. With increasing time, the BM was more corroded, but the LMZ still showed corrosion resistance (Figure 12b–d). After 1 h and 45 min, a slight corrosion effect started to appear on the LMZ boundary (Figure 12e), and when the time was increased to 3 h and 45 min, more corrosion occurred at the boundary and in the upper zone of the LMZ, as pointed out in Figure 12f. In AA7075-ScZr and AA7075-TiB, the LMZ had very good corrosion resistance compared to the standard alloy; this is because of the uniform distribution of the elements, and the effect of corrosion was only observed in the BM, shown in Figure 13 and Figure 14. In AA7075-FeNi, the corrosion resistance of the BM was bad (this appears clearly in Figure 15b–d), and the LMZ was severely affected with increasing time (Figure 15e,f) due to the severe liquation effect along the LMZ and the high sensitivity of Fe to corrosion.

## 4. Conclusions

1. AA7075 alloys modified with alloying elements 0.3% Sc + 0.5% Zr, 1% Ti + 0.2% B, and 1% Fe + 1% Ni were produced by a casting process. AA7075-standard contained primary dendrites of an Al-rich solid solution with a uniform structure and average grain size of 272 ± 20 µm. In AA7075-ScZr and AA7075-TiB, the structure changed from coarse-grained dendritic to limitingly fine nondendritic, and the grain sizes of these alloys were decreased to 11 ± 2 µm and 23 ± 2 µm, respectively. In AA7075-FeNi, a coarse structure with an extremely unfavorable morphology close to a needle structure was obtained with an increase in grain size to 316 ± 15 µm. Homogenization annealing was performed to eliminate the non-equilibrium eutectic phases. The best mechanical properties were obtained from the AA7075-TiB alloy in the as-cast and homogenized states due to the effect of Ti as a modifier and grain refining element.

2. After laser melting, the microstructure was changed, and the grain size decreased significantly after the AA7075-Standard was modified with rare earth elements Sc, Zr, Ti, B, Fe, and Ni. Some equiaxed grains were formed in the region near the boundary of the melted zone; above and below this boundary were the equiaxed grain zone and the heat-affected zone (HAZ), respectively. The best structure after laser melting was in AA7075-TiB. The best microhardness values of the LMZs were observed in AA7075-FeNi and AA7075-ScZr compared to the standard alloy.

3. The severe liquation effect appeared in the AA7075-standard and AA7075-FeNi due to the non-equilibrium solidification. Non-uniform elemental distribution occurred, where a high concentration of Zn and Mg was recorded at the region near the boundary between the BM and the LMZ, then the concentration was decreased in the LMZ due to the evaporation effect of Zn and Mg at a high temperature of the laser process. This liquation affected the quality of the laser-melted area and decreased its corrosion resistance. In case of the modified alloy with Sc + Zr and Ti + B, the LMZs had better corrosion resistance than the other two alloys because of the uniform distribution of elements due to the heterogeneous nucleation and growth of the crystallizing phases in the presence of the many crystallization centers. The corrosion effect was only observed at the region near the boundary between the BM and the LMZ due to the evaporation effect of Zn and Mg at high temperature of the laser process.

4. In future work, the welding parameters will be adjusted to obtain a better microstructure without the formation of hot cracks, and another technique will be used to decrease the solidification range to make these modified alloys suitable for additive manufacturing technology.

## Figures and Tables

**Table 1:** Chemical compositions of the investigated alloys, in wt %.

Alloy	Chemical Composition, wt %												
	Al	Zn	Mg	Cu	Cr	Mn	Ti	B	Fe	Ni	Zr	Sc	Other Elements
AA7075-standard	Bal.	6.8	2.2	1.4	0.3	0.3	-	-	-	-	-	-	<0.1
AA7075-ZrSc	Bal.	6.8	2.2	1.4	0.3	0.3	-	-	-	-	0.5	0.3	<0.1
AA7075-TiB	Bal.	6.8	2.2	1.4	0.3	0.3	1	0.2	-	-	-	-	<0.1
AA7075-FeNi	Bal.	6.8	2.2	1.4	0.3	0.3	-	-	1	1	-	-	<0.1

**Table 2:** Ultimate tensile strength (UTS) results of the investigated alloys in as-Cast and homogenized conditions.

Alloy	UTS, (MPa)	YS, (MPa)	Elongation, % *
As-Cast AA7075-standard	161 ± 6	126 ± 3	1.2
Homogenized AA7075-standard	146 ± 1	81 ± 20	8.3
As-Cast AA7075-ZrSc	289 ± 3	224 ± 20	12.7
Homogenized AA7075-ZrSc	199 ± 10	122 ± 30	8.6
As-Cast AA7075-TiB	293 ± 1	232 ± 9	6.3
Homogenized AA7075-TiB	269 ± 6	169 ± 9	8.3
As-Cast AA7075-FeNi	224 ± 8	194 ± 19	1.7
Homogenized AA7075-FeNi	185 ± 6	161 ± 3	6.6

* Range is less than 0.5.
